# A Case of Dropped Head plus Syndrome

**DOI:** 10.4021/jocmr2009.09.1260

**Published:** 2009-10-16

**Authors:** Aiesha Ahmed, Charles S Specht, Kevin Scott

**Affiliations:** a Penn State College of Medicine, Hershey Medical Center, Department of Neurology EC037, 30 Hope Drive, Hershey, PA 17033, USA; b Penn State College of Medicine, Hershey Medical Center, Department of Pathology, 500 University Dr, Hershey, PA 17033, USA

## Abstract

A patient developed neck extensor weakness along with symmetrical, mild proximal extremity weakness. Electrodiagnostic findings were minimal. Subsequently muscle biopsy was undertaken which showed myopathic changes as well as rimmed vacuoles. This case highlights the discussion of histopatholgical findings seen in neck extensor weakness when it occurs in isolation as well as when it is associated with weakness in other distribution.

**Keywords:**

Neck extensor weakness; Rimmed vacuoles; Myopathy

## Case Report

A 55-year-old right-handed woman presented with chief complaint of neck extension weakness which had been slowly progressive over the past 5 months. She denied pain or focal weakness otherwise. Her neurological review of systems was also negative.

Her past medical history was significant for insulin-dependent diabetes mellitus, diabetic polyneuropathy, rheumatoid arthritis, asthma, COPD, and coronary artery disease. There was no family history of neuromuscular disease. Social history was unremarkable. Medications included Allopurinol, Isosorbide, Clonazepam, Prevacid, Plavix, and Zocor.

Neurological examination showed intact mental status and normal speech. Cranial nerve exam was unremarkable. Neck exam showed 4-/5 (MRC scale) neck extension strength. Power was otherwise full with the exception of mild 4+ to 5-/5 proximal weakness of the deltoids and hip flexors thought as secondary to deconditioning and general poor health. Reflexes were absent throughout. Sensory exam showed a symmetrical deficit to all modalities distal to mid-calf bilaterally. Coordination was intact. Gait was normal.

Laboratory analysis, including complete metabolic profile, CBC, ANA, ANCA, CPK, Anti-Jo, immunofixation, B12, acetylcholine receptor antibodies, and thyroid function studies were normal. Sedimentation rate was elevated at 58.

EMG/nerve conduction study of left upper and lower extremities to include 3 Hz of repetitive stimulation studies showed a moderate sensorimotor polyneuropathy with mixed axonal and demyelinating features secondary to the patients diabetes. Mild active denervation was also seen in the cervical paraspinal muscles.

**Figure 1:**
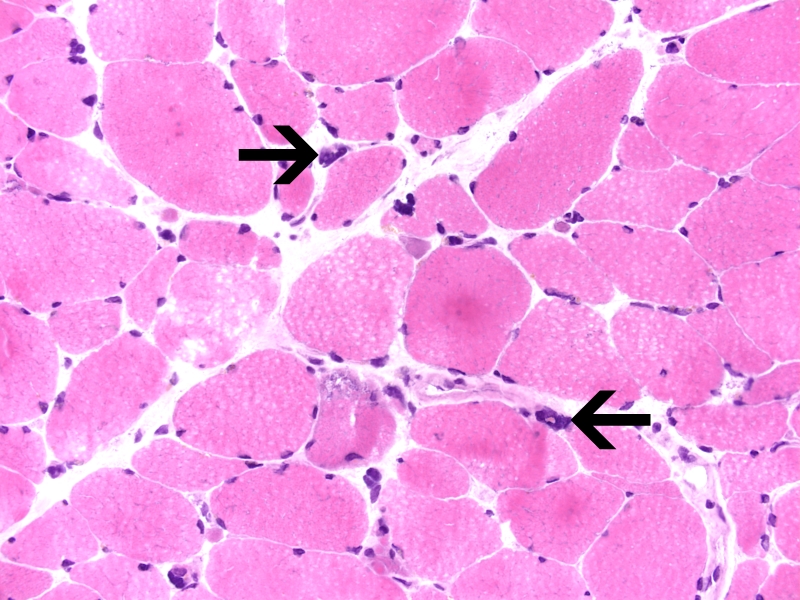
Clumped myofiber nuclei (arrows) and angular-atrophic myofibers suggest a component of denervation atrophy. Note rimmed vacuole with blue granules (arrowhead). Hematoxylin-Eosin, 400x.

**Figure 2:**
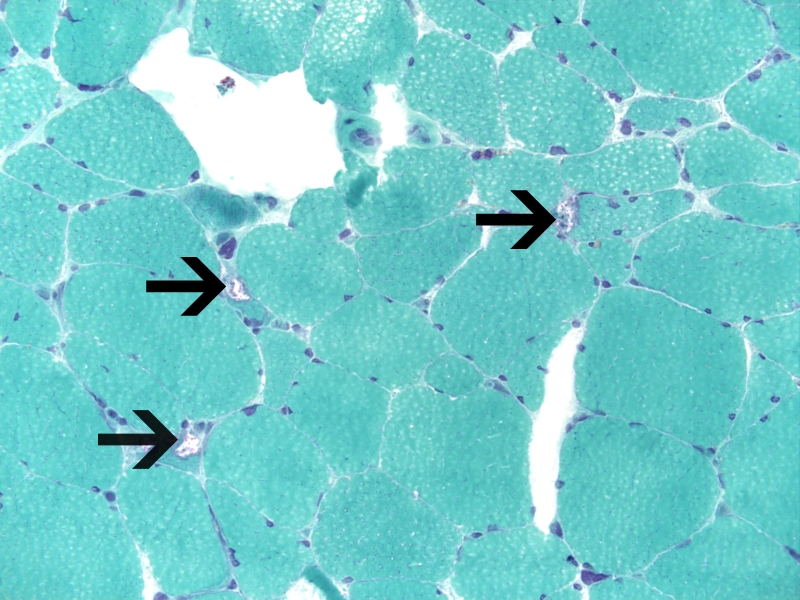
Note rimmed vacuoles with red (fuchsinophilic) particles (arrows). Gomori trichrome, 400x.

**Figure 3:**
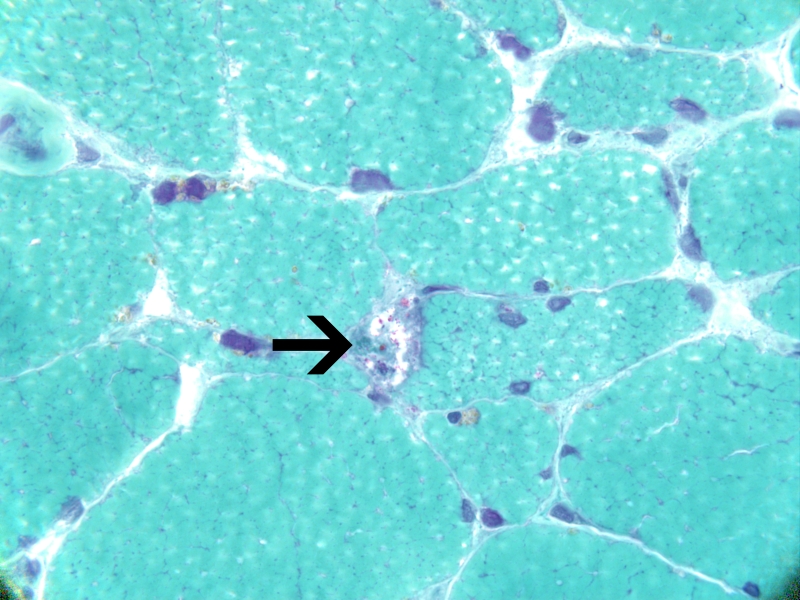
Rimmed vacuole in myofiber, with red (fuchsinophilic) particles. Gomori trichrome, 1000x.

Imaging studies to include MRI of brain, cervical, thoracic, and lumbar spine were unremarkable. Muscle biopsy was performed on right vastus lateralis muscle (biopsy of the cervical paraspinals biopsy could not be pursued due to body habitus). The biopsy revealed moderate variation in myofiber size and shape, with scattered myofiber nuclear clumps. Sections stained for ATPase demonstrated moderate, non-selective, atrophy of type 1 and type 2 myofibers. There were scattered degenerating myofibers with rimmed vacuoles and a few regenerating myofibers were seen. Electron microscopy did not reveal tubulofilamentous material in the rimmed vacuoles. A microscopic focus of perivascular and endomysial lymphocytic infiltrate was noted. Diagnostic increase in MHC1 (inflammation marker) expression was not identified. Features diagnostic for vasculitis were not identified. The rimmed vacuoles were highlighted with the GTC stain; ragged-red fibers were not identified. The methods for phosphorylase, acid phosphatase, phosphofructokinase, and myoadenylate deaminase did not show diagnostic changes. Endomysial fibrosis was not seen (Fig. 1-3).

## Discussion

Neck extension weakness can be a feature of neuromuscular disorders. Even in cases of isolated neck extensor myopathy, there may be additional weakness of periscapular and proximal extremity muscles [1,2] which was seen in our patient as well. Other variations have been reported as well. For example, Rose et al described a patient with dropped head plus syndrome who also demonstrated weakness of bulbar and proximal extremity weakness.

Electrodiagnostic testing results of most patients described as having dropped-head syndrome have reported active denervation in the cervical paraspinal muscles along with myopathic changes in the proximal muscles[2]. Katz et al, however, described myopathic findings and active denervation limited to the cervical paraspinal muscles [1] similar to our patient.

Biopsy of cervical paraspinal and extremity muscles have been described as showing myopathic changes with variability of fiber size, internalized nuclei and fiber splitting [1,2]. Ragged red fibers, accumulation of sarcoplasmic material, increased lipid content, and reduced central NADH staining have been noted inconsistently in cases of isolated neck extensor myopathies[1-3].

Our patient demonstrated mild weakness of proximal muscles in addition to the neck extensor weakness similar to other reports, but demonstrated EMG changes only in the cervical paraspinals. Biopsy showed chronic myopathic changes, but in contrast to the findings described by Katz et al and Liao et al, our patient did not have ragged red fibers, accumulation of sarcoplasmic material, increased lipid content, or reduced central NADH staining. Like Rose et al, our patient also showed inflammatory infiltrate. What is unique about our case is the presence of rimmed vacuoles. To the best of our knowledge, only Morino et al, have reported a patient with neck extensor weakness that developed progressive limb weakness with rimmed vacuoles on biopsy [4]. To our knowledge, our patient is the second case of dropped head plus syndrome with associated rimmed vacuoles seen in the literature. Unlike inclusion body myositis, which can present with neck extension weakness [5], our patient did not have elevated CK levels, electrodiagnostic findings of mixed neurogenic-myopathic pattern, or ultrastructural evidence of tubulofilamentous material in the rimmed vacuoles by electron microscopy.
